# Unlocking Potential and Limits of Kinase Inhibitors: The Highway to Enhanced Cancer Targeted Therapy

**DOI:** 10.3390/pharmaceutics16050625

**Published:** 2024-05-07

**Authors:** Francesca Musumeci, Silvia Schenone

**Affiliations:** Department of Pharmacy, University of Genoa, Viale Benedetto XV, 3, 16132 Genoa, Italy; silvia.schenone@unige.it

Kinases are a family of enzymes comprising over five hundred members, which, when overexpressed or hyperactivated, are implicated in the pathogenesis of numerous hematological and solid cancers. This evidence has spurred extensive research into molecules with targeted anti-cancer activity and led to the approval of the first monoclonal antibody, trastuzumab, in 1998 and the first small-molecule kinase inhibitor, imatinib, in 2001. Since then, the search for new kinase inhibitors has attracted the attention of scientists, as they can provide a powerful approach to mitigating the side effects associated with traditional antineoplastic agents and enabling convenient oral administration in the case of small-molecule inhibitors. The impact of targeted cancer therapy is underscored by the approval of over 70 kinase inhibitors, with approximately 25 new compounds emerging in the past five years [1,2]. Traditionally, small-molecule kinase inhibitors are classified into six types: type I inhibitors, which target the ATP-binding pocket of active kinases; type II inhibitors, which target the ATP-binding pocket of inactive kinases; type III inhibitors, allosteric inhibitors that bind a site adjacent to the ATP-binding pocket; type IV inhibitors, allosteric inhibitors that bind a site distant from the ATP-binding pocket; type V inhibitors, bivalent inhibitors; and type VI inhibitors, covalent inhibitors. Most currently approved kinase inhibitors belong to types I and II. Some examples of other types of kinase inhibitors include trametinib (a type III allosteric inhibitor targeting MEK1/2), asciminib (a type IV allosteric inhibitor targeting Bcr-Abl), and afatinib (a type VI covalent inhibitor targeting EGFR).

The fervent interest in kinase inhibitors from the academic and industrial world points toward a promising expansion of this field in the coming decades. This enthusiasm is driven by the untapped potential of targeting kinases alongside the pressing need to address current limitations.

Notably, despite significant progress, only approximately 20% of the kinome has been targeted to date, indicating vast opportunities for discovering novel treatment options once the roles of additional kinases are elucidated. Furthermore, ongoing research points out allosteric inhibition as a powerful approach to reduce adverse effects and fight resistance to the drug due to mutations in the active site of the enzyme. In this context, developing novel types of allosteric inhibitors, such as compounds targeting pseudokinase domains or extracellular domains, is a promising new route [2,3].

Regarding the current limitations of kinase inhibitors, major issues can be identified in the onset of mutations that confer drug resistance and the general suboptimal pharmacokinetic profiles exhibited by small-molecule kinase inhibitors.

Drug resistance poses a significant challenge to the long-term efficacy of kinase inhibitors, as treatment with most approved drugs is eventually interrupted following prolonged use. Resistance can arise through various mechanisms, with gene mutations being the most prevalent cause. Mutations in the catalytic domain frequently confer resistance to type I and II kinase inhibitors. This evidence encouraged, and is still encouraging, the search for new efficacious approaches [4].

First, the most intuitive strategy plans the design and synthesis of compounds that are also active against resistant isoforms. The approval of ponatinib is a pertinent example, as it is a multitargeted covalent kinase inhibitor that is able to inhibit the T315I Bcr-Abl gatekeeper mutation.

Second, as previously mentioned, the development of allosteric inhibitors constitutes another exciting opportunity to bypass resistance related to mutations in the catalytic domain. In particular, combining an ATP-competitive inhibitor and an allosteric inhibitor often led to improved efficacy compared to the single-agent regimen [3].

Third, the development of antibody–drug conjugates (ADCs) has been highlighted. An ADC comprises a monoclonal antibody and cytotoxic agent attached by a linker. The rationale behind the design of this class of compounds lies in the synergy between the highly specific targeting ability of monoclonal antibodies and the potent cytotoxic activity of the attached agents. Gemtuzumab ozogamicin (Mylotarg^®^) was the first ADC approved by the FDA in 2000. Since then, about 15 ADCs have been released. Notably, Fam-trastuzumab deruxtecan (Enhertu^®^) is an ADC targeting the HER kinase and incorporates the topoisomerase I inhibitor DXd as its cytotoxic agent. It was approved in 2019 as a second-line treatment for patients with unresectable or metastatic HER2-positive breast cancer [5].

Fourth, a recent approach gaining attention is synthetic lethality, which exploits the concept that in certain tumors, the expression of two or more genes is altered, and targeting both synthetic lethal factors enables the selective killing of cancer cells. The most relevant example of synthetic lethality is the use of the poly(ADP-ribose) polymerase inhibitor olaparib in the treatment of breast cancer associated with BRCA1/2 mutations [6]. However, kinase inhibitors also present an opportunity to leverage the concept of synthetic lethality. For instance, cyclin-dependent kinases (CDKs) are being investigated as synthetic lethal players when combined with specific oncogenes, such as MYC, TP53, and RAS [7].

At the same time, the poor suboptimal pharmacokinetic profile generally associated with kinase inhibitors has prompted the search for efficient drug delivery systems. In this frame, nanoformulations offer an effective tool to improve the absorption, distribution, metabolism, and excretion (ADME) properties and, consequently, the bioavailability of kinase inhibitors. Nanocarriers include liposomes, micelles, gold and polymeric nanoparticles, bovine serum albumin, and metal–organic frameworks. Notably, the nanoparticle surface can be functionalized with specific ligands, such as antibodies or peptides, capable of recognizing particular proteins on the surface of cancer cells, thus facilitating active targeting. This approach enables the selective release of the drug to the neoplastic cells, thereby enhancing therapeutic efficacy while minimizing off-target effects. Moreover, nanodelivery represents an effective strategy for overcoming resistance mechanisms [8].

The Special Issue features a comprehensive overview of the latest developments in kinase inhibitor research across various fronts, including in silico studies, the synthesis and identification of new compounds, drug delivery methods, overcoming resistance mechanisms, the potential to target specific kinases or use selected inhibitors, and biological and pharmacokinetic evaluations. Herein, an overview of the most innovative articles is reported. Čermáková et al. (contribution 1) provided deep insight into the ability of Bruton’s tyrosine kinase inhibitor zanubrutinib to modulate cancer resistance by inhibiting anthracycline metabolism and efflux, suggesting a new combination therapy including both the drugs. Sunoqrot et al. (contribution 2) proposed vitamin E TPGS-poloxamer nanoparticles to deliver the new PI3Kα inhibitor R19 in breast cancer cell lines. The research carried out by the authors showed that the formulation possessed enhanced activity compared to R19 alone, good cancer cell selectivity, and high biocompatibility, paving the way for clinical translation. Poggialini et al. (contribution 3) performed a study to identify new candidates for glioblastoma (GBM) treatment since this tumor still has a poor prognosis. Starting from the in-house library of pyrazolo[3,4-*d*]pyrimidines, they performed extensive research, including enzymatic assays of Src/Bcr-Abl kinases, in vitro tests on four GBM cell lines, and a deep ADME evaluation that led to the discovery of a compound (namely derivative **5** of the paper) as a suitable candidate for in vivo evaluation. Sabetta et al. (contribution 4) analyzed the effect of dasatinib in 2D monolayer cultures of prostate cancer and glioblastoma cell lines and the corresponding 3D spheroids and 3D bioprinting models. Three-dimensional cell models better resemble tumor complexity associated with drug resistance than two-dimensional models and, for this reason, are gaining increasing attention in preclinical studies. In this work, the authors took a step forward in this field, identifying that the 3D bioprinted model utilizing an alginate–gelatin hydrogel was endowed with improved feasibility, reproducibility, and scalability than the classical 3D spheroid model.

Overall, this Special Issue offers a panoramic view of the current state of the field. Each contribution showcases the multifaceted approaches researchers are employing to unlock the power of kinase inhibition for cancer treatment. These studies contribute valuable data to the scientific community and pave the way for translating the findings into clinical applications.
